# Polymeric Scaffolds for Dental, Oral, and Craniofacial Regenerative Medicine

**DOI:** 10.3390/molecules26227043

**Published:** 2021-11-22

**Authors:** David T. Wu, Jose G. Munguia-Lopez, Ye Won Cho, Xiaolu Ma, Vivian Song, Zhiyue Zhu, Simon D. Tran

**Affiliations:** 1 Craniofacial Stem Cells and Tissue Engineering Laboratory, Faculty of Dentistry, McGill University, Montreal, QC H3A 0C7, Canada; DavidT_Wu@hsdm.harvard.edu (D.T.W.); jose.munguia-lopez@mail.mcgill.ca (J.G.M.-L.); xiaolu.ma@mail.mcgill.ca (X.M.); vivian.song@mail.mcgill.ca (V.S.); 2 Department of Oral Medicine, Infection, and Immunity, Harvard School of Dental Medicine, Harvard University, Boston, MA 02115, USA; yewon_cho@hsdm.harvard.edu; 3 Harvard John A. Paulson School of Engineering and Applied Sciences, Harvard University, Cambridge, MA 02138, USA; 4 Wyss Institute for Biologically Inspired Engineering, Harvard University, Boston, MA 02115, USA; 5 Department of Bioengineering, Faculty of Engineering, McGill University, Montreal, QC H3A 0E9, Canada; zhiyue.zhu@mail.mcgill.ca

**Keywords:** polymers, polymeric scaffolds, tissue engineering, regenerative medicine, bone regeneration, sinus augmentation, periodontal regeneration, pulp regeneration, whole tooth regeneration, salivary gland regeneration

## Abstract

Dental, oral, and craniofacial (DOC) regenerative medicine aims to repair or regenerate DOC tissues including teeth, dental pulp, periodontal tissues, salivary gland, temporomandibular joint (TMJ), hard (bone, cartilage), and soft (muscle, nerve, skin) tissues of the craniofacial complex. Polymeric materials have a broad range of applications in biomedical engineering and regenerative medicine functioning as tissue engineering scaffolds, carriers for cell-based therapies, and biomedical devices for delivery of drugs and biologics. The focus of this review is to discuss the properties and clinical indications of polymeric scaffold materials and extracellular matrix technologies for DOC regenerative medicine. More specifically, this review outlines the key properties, advantages and drawbacks of natural polymers including alginate, cellulose, chitosan, silk, collagen, gelatin, fibrin, laminin, decellularized extracellular matrix, and hyaluronic acid, as well as synthetic polymers including polylactic acid (PLA), polyglycolic acid (PGA), polycaprolactone (PCL), poly (ethylene glycol) (PEG), and Zwitterionic polymers. This review highlights key clinical applications of polymeric scaffolding materials to repair and/or regenerate various DOC tissues. Particularly, polymeric materials used in clinical procedures are discussed including alveolar ridge preservation, vertical and horizontal ridge augmentation, maxillary sinus augmentation, TMJ reconstruction, periodontal regeneration, periodontal/peri-implant plastic surgery, regenerative endodontics. In addition, polymeric scaffolds application in whole tooth and salivary gland regeneration are discussed.

## 1. Introduction

Tissue deficiencies of the dental, oral, and craniofacial (DOC) structures can result from numerous diseases, disorders, and injuries, including infections, genetic disorders, cancers, and trauma. According to the Global Burden of Diseases, Injuries, and Risk Factors Study published in 2017 (GBD 2017), oral disorders combined for the greatest age-standardized prevalence and incidence in the world [1].

Recent advances in biomaterials and manufacturing techniques have enabled the development of various types of materials including natural and synthetic polymeric scaffolding materials for clinical applications for the repair and regeneration of various deficiencies and deformities in DOC structures [2]. With a focus on understanding the inherent properties of a biomaterial at the biological interface, various tissue engineering strategies and surgical therapies have been developed to be translated into the clinical arena in order to successfully restore both tissue morphology and function. Largely, the clinical usage of polymeric scaffolds with or without additional cellular or biologic mediators are well-documented in regenerative therapies of tooth structures, supporting periodontal apparatus, alveolar bone, maxillary sinus, temporomandibular joint, and salivary glands.

Based on recent literature, this review presents an overview of key polymeric scaffolds used in dental, oral, and craniofacial regenerative medicine including their properties, benefits, drawbacks, and clinical applications.

## 2. Overview of Polymeric Scaffold Materials in DOC Regenerative Medicine

**Figure 1:**
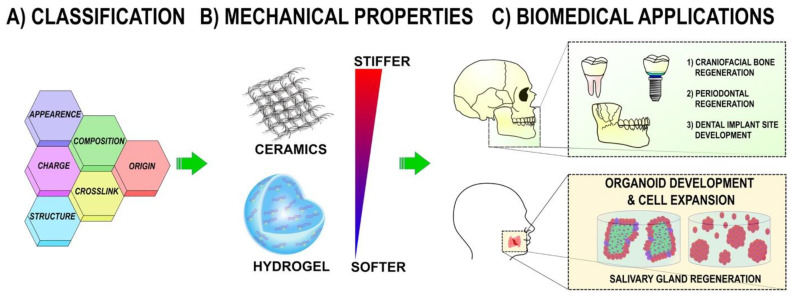
Polymeric Scaffolds for Dental, Oral and Craniofacial Regeneration. (**A**) Polymeric scaffolds can be classified according to their appearance, charge, structure, composition, crosslinking and origin. (**B**) Polymeric scaffolds have a wide range of mechanical properties that can be tuned to affect cellular behavior. (**C**) Polymeric scaffolds have various applications in tissue engineering in the context of dental, oral, and craniofacial regeneration.

For decades, biomaterials have been extensively studied in biomedical applications. Polymeric materials and polymeric films significantly impact dentistry applications used as antimicrobial films and scaffolds (for cell expansion or a transplant, for instance) [3]. Polymers are organic materials composed of long chains of atoms joined by covalent bonds that can be naturally derived or synthetic [4]. The ideal polymer for tissue engineering should be (1) mechanically stable, (2) biocompatible and bioactive, and (3) biodegradable (Figure 1).

First, mechanical properties are crucial in the development of new biopolymers. A scaffold should provide the stiffness of the tissue origin, ensuring its native mechanical properties. Considering that different tissues require specific mechanical characteristics due to multicellular composition, it is ideal to create tunable polymeric matrices that allow mechanical changes inside of the construct through time [5].

Second, biocompatibility means cells or tissue can survive when interacting with polymer, but not necessarily for them to duplicate or differentiate. Some biocompatible polymers support cell viability, but cells are not able to self-duplicate, differentiate, or migrate. This results in cell death within short period of time. In this context, polymeric material must be both biocompatible and bioactive. When a polymer is bioactive, in general, it is biocompatible and allows cell attachment, migration, differentiation, proliferation, and the cell can perform its biological functions [6].

Last, biomaterial should be biodegradable in a short (one month for soft tissue, i.e., salivary glands) or a long time (six months to one year for hard tissue like bone). In soft tissue, this will allow the host to reabsorb the material once an artificially regenerated tissue or organoid has been implanted, driving the interaction of artificial and host environments. For hard tissue such as bone, the calcification process takes time, thus the polymeric scaffold should provide mechanical support for a longer period, ensuring the host could accept the new implant and allowing host cells to interact with the new, engineered graft [7].

### 2.1. Natural Polymers

The most common carbohydrates-based biopolymers used for hydrogels are cellulose, chitosan, and alginate. From natural source polymers, protein-based hydrogels are attractive due to the bioactive molecules allowing cell attachment. Some of the most popular biopolymers used include collagen, gelatin, fibrin, and laminin. This section provides an overview of the various natural polymers used in DOC regenerative medicine. The advantages and drawbacks of these natural polymers are summarized (Table 1).

#### 2.1.1. Alginate

Alginate is a natural polymer isolated from algae [8]. This polymer is comprised of 1-4-*β*-d-mannuronic acid (M) and α-l-guluronic acid (G), where the organization and number of G and M blocks drive the formation of an “egg-box” by adding divalent ions allowing to produce a stable and stiffer three-dimensional (3D) hydrogel [9]. Alginate can be modified by incorporating the adhesion ligand (arginine-glycine-aspartic acid (RGD)) that promote cell attachment. Alginate is an excellent biomaterial that can be used to confer specific cellular interactive properties allowing for the control of long-term gene expression of cells encapsulated within the hydrogel [9]. Alginate hydrogels can be crosslinked with metallic ions such as calcium, and can also be engineered to be enzymatically degradable by cleavage enzymes produced by the encapsulated and surrounding cells [10]. Alginate hydrogels can be designed with tunable mechanical properties such as stiffness and stress relaxation to regulate stem cell fate and activity [11].

#### 2.1.2. Cellulose

Cellulose is one of the most abundant polymers in nature, extracted from plants’ cell walls and produced by certain bacteria [12,13]. However, its water-insoluble nature makes it difficult to manipulate; its water-soluble derivatives can be obtained by etherification. Cellulose is comprised of d-glucopyranose units linked by *β*-1,4-glycosidic bond, presenting abundant hydroxyl groups that can be used as moiety molecules to prepare hydrogels by physical crosslink [13,14].

#### 2.1.3. Chitosan

Chitosan is the second most abundant polysaccharide in nature, and it is obtained from deacetylated chitin, which is isolated from the exoskeletons of crustaceans, fungi, and insects [15]. Chitosan is a linear polymer composed of glucosamine and *N*-acetyl glucosamine units linked by *β*-1,4-glycosidic bond, with free amino groups that provide stronger reactivity and greater solubility than chitin [16]. Both chitin and chitosan can form hydrogels due to a large number of functional groups (hydroxyl and/or amine groups) available for chemical reactions [15,16,17,18,19].

#### 2.1.4. Silk

Silk is another natural polymer used in tissue engineering of bone, cartilage, tendon and ligament tissues. Silks are fibrous proteins produced by silkworms and spiders with remarkable mechanical properties [20]. Silk can be chemically modified to immobilize growth factors and adhesion factors. In addition, silk can be genetically tailored to allow for production of recombinant silk that can be applied for cellular targeting and drug delivery [21]. Silk-based biomaterials have been studied in vitro and in vivo, in wound healing and regenerative medicine [22].

#### 2.1.5. Collagen

In mammalian organisms, collagen is the most abundant protein representing 25% (dry weight) of total proteins. This protein is expressed by cells from the skin, ligament, cartilage, tendon, and bone, representing the main structural element in these connective tissues [23,24]. Among all collagen types, type I collagen is the most used to prepare hydrogels. The triple-helical configuration of the collagen’s chain allows it to self-assemble at physiological temperature and pH (37 °C and pH ≈ 7.2, respectively) to form a stable 3D structure, while at 4 °C it remains in a liquid state. Collagen is rich in RGD adhesion ligand, which enables cell-biomaterial interaction leading to cell adhesion [24,25]. The collagen′s mechanical properties can be tuned by using different crosslinked agents [24]. One benefit of collagen is that patients′ cells and enzymes can readily degrade and remodel these materials. In addition, collagen can be processed into micro or nanoparticles can be applied as delivery vehicle for other biological components such as biologics and growth factors [4].

#### 2.1.6. Gelatin

Gelatin is obtained by the acid or alkaline hydrolysis process of collagen [26]. This protein presents the opposite behavior to collagen in gelation; temperatures lower than 25 °C allow gelatin gels to form, while physiological temperature (37 °C) leads to a liquid state. The sol-gel transition occurs at ≈30 °C. The addition of chemical components such as genipin or chemical modification in the gelatin’s structures (i.e., methacrylate) provides stability of the 3D structure at physiological conditions. Gelatin can form copolymer with alginate and benefit from the desired properties of both individual materials applicable to the osteogenic differentiation of adipose-derived stem cells in 3D [27].

#### 2.1.7. Fibrin

Fibrin is a natural tissue sealant and extracellular matrix (ECM) component. The easy way to form fibrin hydrogels is by combining fibrinogen and thrombin at 37 °C, where the ratio of these two components can tune the mechanical properties of the gel, regulating the thickness of the internal fibers and the porosity of the hydrogel [28,29,30]. Fibrinogen is a 45-nm length dimeric glycoprotein made up of three pairs of distinct chains, Aα, Bβ, and γ chains. The fibropeptides A (FPA) and B (FPB) are the ones involved in the polymerization of the fibrin hydrogels when exposed to thrombin [30].

#### 2.1.8. Laminin

Laminins are another important ECM proteins that play an essential role in ECM architecture, cell adhesion, and molecules binding [31]. Laminins are basement membrane multimeric glycoproteins formed from three chains α, β, and γ, and there are sixteen isoforms with tissue-dependent distribution [31]. In general, laminins are incorporated into synthetic of biological hydrogels to recapitulate the dynamic nature and biological complexity of nerve niches [32,33]. Recently these laminin-based hydrogels have been used as 3D platforms for salivary gland regeneration [29].

#### 2.1.9. Decellularized Extracellular Matrix (dECM)

Decellularized ECM (dECM) has gained more popularity in using it as hydrogels for tissue regeneration, in particular for organoid formation in vitro [34]. This complex network of macromolecular substances has a crucial role in cell adhesion, migration, differentiation, and functional expression regulating tissue development and homeostasis [35]. Matrigel^®^ is one of the commercial and most common ECM derived from mouse tumors, which secrete abundant basement membrane proteins such as laminin, collagen IV, entactin, heparan sulfate, etc. Matrigel^®^ is liquid at 4 °C, and forms an irreversible gel with an increment in temperature (37 °C) [36]. dECM has been isolated from different tissue sources, including human, porcine, bovine, mouse among others, by mechanical, chemical and/or enzymatical process [37,38]. Generally, the dECM gels can be formed by temperature, salt ion concentration, and pH change or by the addition of crosslinking agents [35].

#### 2.1.10. Hyaluronic Acid (HA)

The precise chemical structure of hyaluronic acid (HA) contains repeating units of d-glucuronic acid and *N*-acetyl-d-glucosamine [39]. HA is classified as a non-sulfated glycosaminoglycan and is the main constituent of the ECM of connective tissue, synovial fluid, and other tissues. It possesses various physiological and structural functions, including cellular interaction, interactions with growth factors and regulation of the osmic pressure. All of these functions help to maintain the structural and homeostatic integrity of the tissue [40,41]. HA has shown anti-inflammatory, anti-edematous, and anti-bacterial effects for the treatment of periodontal disease.

**Table 1:** Advantages and disadvantages of natural polymers for dental, oral and craniofacial regenerative medicine.

Polymer	Advantages	Disadvantages	Reference
Alginate	Biocompatible & biodegradable Tunable Mechanical Properties Low cost of production	Lack of bioactivity Low mechanical strength Rapid degradation rate	[8,9,11]
Cellulose	Contain 3D porous structure Allow for cell adhesion Tunable chemical, physical and mechanical properties	Water insoluble Not biodegradable in humans	[14]
Chitosan	Biocompatible Hydrophilic structure promotes cell adhesion, proliferation and differentiation	Costly production Inconsistent properties Environmentally unfriendly	[18,19]
Silk	Remarkable mechanical properties Chemically modifiable to include cell adhesion and growth factors	Ecological concerns	[20,22]
Protein-Based(Fibrin, collagen, laminin)	Tissue regenerative Ability to convert bioinert scaffold into bioactive scaffold as coating material	Possible immunogenicity and allergenicity	[28,31,35]
dECM	Tissue regenerative Autologous	Immune response from cellular DNAs	[34]
Hyaluronic Acid	Bioactive and biocompatible Versatile for various applications after chemical modifications	Poor mechanical properties Rapid degradation in vivo	[41]

### 2.2. Synthetic Polymers

Synthetic polymers have been widely used for different biomedical applications. Some of the most common synthetic polymers used in tissue engineering are polylactic acid (PLA), polyglycolic acid (PGA), polycaprolactone (PCL), and polyethylene glycol (PEG) [4,42,43]. The mechanical properties of synthetic polymers make them an attractive material for different biomedical purposes. However, the lack of bioactive components (limited cell anchoring sites) on synthetic polymer poses a significant challenge for tissue engineering as cells cannot readily proliferate, differentiate, or migrate. The chemical modification of synthetic polymers allows the incorporation of bioactive molecules to produce biocompatible and functional materials that ensure cell biology performance like the native environment.

#### 2.2.1. Polylactic Acid (PLA)

PLA is a good candidate polymer scaffold for DOC tissue engineering. PLA undergoes hydrolytic degradation to form soluble lactic acid naturally present in the human body [4]. PLA can be combined with other degradation resistant polymers such as PEEK to fabricate multi-material scaffolds via selective laser sintering (SLS) to enhance scaffold bioactivity, biocompatibility, and cytocompatibility [44]. PLA can also be blended with PCL with 3D electrospinning technique to enhance mechanical properties, bioactivity and osteogenic differentiation [45].

#### 2.2.2. Polyglycolic Acid (PGA)

PLGA, a co-polymer of lactic acid and glycolic acid, has tunable degradation rate depending on the ratio of lactic acid to glycolic acid in the copolymer due to the difference in hydrophilicity of the two monomers [46]. Several PGA-based polymers were used and compared for in vitro tissue engineering including PGA-PLA, PGA-PCL, and PGA-poly-4-hydroxybutyrate (P4HB). PGA-PLA and PGA-P4HB demonstrated enhanced tissue formation compared to PGA-PCL scaffolds. This may be attributed to achieving a balance between the rate of scaffold degradation and tissue formation for maintaining mechanical integrity of the replacement tissue [47].

#### 2.2.3. Polycaprolactone (PCL)

PCL has high mechanical strength and can be used as polymeric scaffolds for bone and periodontal tissue engineering [48,49]. However, it undergoes very slow hydrolytic degradation in vivo, thus may not be ideal for certain clinical indications where fast polymeric scaffold degradation is desired. PCL lacks features that promote cell-adhesion. Nevertheless, its hydrophobicity and surface properties can be modified by polydopamine coating to improve cell and therapeutic protein adhesion and serve as sites for hydroxyapatite nucleation and mineralization [49].

#### 2.2.4. Polyethylene Glycol (PEG)

PEG and derivates have been extensively used as scaffolds or injectable hydrogels. Lu et al. created an injectable hydrogel comprised of PEG diacrylate (PEG-DA) and fibrinogen as a scaffold for dental pulp tissue engineering [50]. The concentration of PEG-DA modulated the mechanical properties of the hydrogel. The hydrogels showed cytocompatibility with dental pulp stem cells (DPSCs), where cell morphology, odontogenic gene expression, and mineralization were influenced by the hydrogel crosslinking degree and matrix stiffness [50].

#### 2.2.5. Zwitterionic Polymers

Given their unique material properties, zwitterionic polymers have shown promising results as tissue scaffolds for regenerative medicine and as drug delivery vehicles [51]. By definition, a zwitterionic polymer has both a positive and a negative charge. In nature, proteins and peptides are examples of such polymers. Their 3D structure is therefore determined by their charge distribution. This property can be used to design synthetic polymers of the desired 3D structure by polymerizing charged zwitterionic monomers or by making modifications after polymerization [52].

Thanks to the electrostatic interactions, they are capable of forming hydration shells. This characteristic makes zwitterionic polymers great antifouling materials [53]. In a study done in 2019, Jain exploited the low fouling characteristic of polycarboxybetaine (PCB) polymers along with carboxybetaine disulfide cross-linker (CBX-SS) that facilitates degradation. The cross-linked PCB/CBX demonstrated excellent non-fouling properties and degradability, making it a promising material for future tissue engineering and drug delivery [54].

As the distribution of charges along the polymer differs, they can display neutral, anionic, or cationic characteristics. Under different environments, they can behave as antipolyelectrolyte or polyelectrolyte [52]. Factors such as pH and temperature are stimuli to the polymer to modify its behavior. Using zwitterionic materials, researchers were able to achieve more precise drug delivery. In one study, crosslinked l-glutamic acid (E) and l-lysine (K) polypeptides hydrogels allowed for drug release and enzymatic degradation in the presence of trypsin, an inflammatory marker. The release is also responsive to pH due to the interactions of the charges [51].

Although zwitterionic hydrogels have good biocompatibility and respond to stimuli, they lack mechanical strength to be an ideal tissue supporting material in direct contact with blood. This problem was solved in a study done in 2020 by combining Electrospun fiber scaffold to zwitterionic hydrogels to achieve biocompatibility and mechanical strength to make long-term blood contact devices possible [55].

The combination of its great hemocompatibility, superior non-fouling properties, charge-switching abilities, and resistance to protein adsorption make zwitterionic polymers very attractive in regenerative medicine in the last decade, specifically in bone regeneration and craniofacial tissue engineering [54]. Zwitterions have also been used in a variety of other different clinical applications, such as in medical implants, dressings and tissue scaffolds for wound healing, drug delivery caries, and biosensors.

Advancements in regeneration of craniofacial tissues such as bone, cartilage, muscle, skin, PDL, and mucosa have been based on finding new ways to enhance and optimize tissue engineering procedures while limiting negative side effects [56]. Specifically, the field of bone tissue engineering constantly aims to find smarter scaffolds capable of avoiding severe side effects associated with bone regeneration treatments by minimizing the administration dosage. A recent study published in 2020 by Liu et al. combining PLGA scaffolds with zwitterionic PSBMA found that this novel biodegradable composite scaffold resulted in successful bone healing at an ultra-low dose [57]. The capacity of zwitterionic polymers to maintain protein bioactivity in the preparation of scaffolds showcases this material’s potential to improve the efficiency of bioactive drug therapies. Furthermore, it was found that certain surface modifications of vascular grafts, like the addition of zwitterionic polymers, enhanced hemocompatibility which prevented thrombosis on artificial vascular grafts [58]. Zwitterionic hydrogels have also shown efficacy as drug delivery systems [51]. These promising features of zwitterionic polymers are the reason behind its increasing popularity in regenerative medicine.

### 2.3. Bioceramics

Given the thermal and chemical stability of ceramics, their high strength, wear resistance and durability, bioceramics have found broad applications in hard tissue repair, such as bone and teeth [59]. Bioceramics fulfill a unique function as biomedical materials as with proper material selection and fabrication, they can be bioinert, bioactive which can have interfacial interactions with surrounding tissues, or biodegradable [60].

Synthetic hydroxyapatite (HA) is bio-resorbable and can form strong chemical bonding with bone in vivo by increasing the local concentration of Ca^2+^ [61]. HA can be employed in forms of powder etc. to fill bone defects; this bone filler scaffold can encourage a rapid filling of the void by integrating bone structures and supporting bone in growth. But its poor fatigue properties render it to be unsuitable for long-term load bearing applications.

Calcium phosphate compounds are also resorbable and their dissolution products can be assimilated by the human body [62]. Beta-tricalcium phosphate (*β*-TCP) is a promising material for bone regeneration applications due to its biocompatibility, osteoconductivity and osteoinductivity properties [63,64]. In periodontal and alveolar bone regeneration, *β*-TCP is often used as part of a composite graft. One example is its usage with HA to create a novel biomimetic material to regenerate bone. *β*-TCP can dissolve in the presence of acids released by cells such as osteoclasts or macrophages [65].

Bio-Oss is a deproteinized bovine bone which has been used in dentistry for bone augmentation due to its osteoconductive properties [66]. The material has shown its effectiveness, safety, and high success rates regarding the quality and quantity of bone formation in grafting procedures. One clinical benefit is the elimination of the need to harvest autogenous bone. In the sites grafted with Bio-Oss, newly formed woven and lamellar bone can be found with an intimate interface with the Bio-Oss graft particles.

## 3. Clinical Applications

### 3.1. Craniofacial and Alveolar Bone Regeneration

Surgical periodontal therapies involving regeneration of alveolar bone, cementum and periodontal apparatus are widely used to achieve adequate bone volume and attachment level. Indications for hard tissue and periodontal attachment regeneration include ridge preservation after tooth extraction, treatment of bony defects (i.e., fenestrations, dehiscence, horizontal and vertical defects), and pre-prosthetic surgery prior to or at the time of implant placement. To achieve morphological and functional repair of alveolar bone and supporting periodontal apparatus, a variety of surgical modalities are available for bone augmentation such as autologous grafts, bone substitute materials, natural and synthetic scaffolds, biologics, etc.

Autologous bone materials contain a mature bony matrix, viable bone cells, mesenchymal stem cells, endothelial cells, growth factors, and cytokines. Together, these components provide osteogenic, osteoconductive and osteogenic properties, making autografts an excellent filling material to regenerate larger bony defects. Donor sites can be either intraoral or extraoral: mandibular ramus and symphysis, maxillary tuberosity, tori and exostoses, tibia or iliac crest. For many decades, autologous bone grafting remained the gold standard of augmenting edentulous areas. However, its recent comparisons with non-autologous bone substitute materials reveal effective ways to avoid additional complications associated with autologous bone harvesting [67].

Commonly used allogeneic bone materials include demineralized freeze-dried bone allograft (DFDBA) and freeze-dried bone allograft (FDBA) derived from human cadavers. DFDBA offers osteoinductive and osteoconductive potential; FDBA offers osteoconductive potential with a slower resorption rate [68,69]. The decalcifying process of FDBA makes bone morphogenetic proteins (BMP) available for effective bone regeneration [70], while the freeze-drying process lowers the antigenicity. Bone turnover and integration of FDBA or DFDBA at recipient site can be improved by directly administering biological mediators like platelet-rich growth factors (PDGF), BMP, enamel matrix derivatives (EMD), vascular endothelial growth factor (VEGF), and fibroblast growth factor (FGF) at the time of augmentation [68,71,72].

Xenogeneic bone substitute materials are of porcine or bovine origin containing anorganic bone matrix. Alloplastic bone fillers include hydrogels of HA and bioactive ceramics like β-tricalcium phosphate (β-TCP), calcium sulphate hemihydrate, and hydroxyapatite, which are often selected in tissue engineering for their biocompatibility, ease of handling, and similar structural and chemical composition of natural bone [73]. HA also offers antimicrobial and anti-inflammatory effects that protect the filler material from bacterial colonization [74]. The main goal of using xenogeneic or alloplastic substances is to provide an osteoconductive matrix for new bone formation to fill critical-size defects. Successful delivery and release of osteogenic and osteoinductive mediators as well as upregulation of pro-angiogenic factors were also observed in the usage of bovine bone, HA, and calcium phosphate as carriers [68,75,76].

Collagenous barriers are the most frequently selected scaffold in hard tissue regeneration for their superior biocompatibility and direct effects on bone formation, periodontal ligament (PDL) formation, and gingival fibroblastic activity. This natural protein-based polymer can be obtained from pericardium, skin, and tendons of human, porcine or bovine sources [68,77,78]. The degradation of resorbable natural scaffolds is driven by rapid enzymatic breakdown by macrophages and polymorphonuclear cells, or bacterial collagenases. On the other hand, non-resorbable membranes require a second surgery to retrieve them, which can lead to increased rates of post-operative complications and contamination with bacteria. Examples include expanded polytetrafluoroethylene (e-PTFE), high-density polytetrafluoroethylene (d-PTFE), titanium-reinforced polytetrafluoroethylene, and titanium mesh [71].

Synthetic polymer scaffolds include synthetically constructed PGA, PLA, polyesters, and co-polymers, which consist of aliphatic polyesters [79]. Though they do not have any inherent pro-angiogenic properties, synthetic polymers serve as effective carriers for delivering pro-angiogenic agents via controlled release. Currently available resorbable examples for maxillofacial and dental surgery include PGA and PLA scaffolds which are degraded via hydrolysis to lactic and glycolic acid, respectively and then metabolized to CO_2_ and H_2_O in Krebs cycle [71,80]. The usage of biodegradable synthetic polymers proposes an alternative to other grafting techniques and their associated complications such as a secondary retrieval surgery in non-resorbable membranes, donor site morbidity in autografts, and host immune-mediated graft rejections in allografts [81]. Furthermore, biological, and physical properties of synthetic polymers can be enhanced by adding an inorganic component. Examples include hydroxyapatite, calcium nitrate, TCP, and bioactive glasses [81]. These modifications serve to improve the osteointegration of the polymer by promoting contact with native bone and controlling the degradation rate. In a similar manner, antibiotics and medications can be delivered via synthetic scaffolds to reduce infections at the recipient site [81,82,83].

In addition to grafting, biologic agents and cellular therapy can be employed to accelerate the bone remodeling process and enhance osteogenic, osteoconductive and/or osteoinductive potential of augmentation procedures. However, the high cost of biologics remains a challenge in clinical usage.

In this section, the clinical applications of bone substitute materials (autografts, allografts, alloplasts, and xenografts) and barrier scaffolds (natural and synthetic) will be discussed in the context of alveolar ridge preservation, vertical and horizontal ridge augmentation, maxillary sinus augmentation, and periodontal regeneration will be discussed. The parameters of selecting surgical modalities are dictated by physicochemical, mechanical and biological properties of polymers for tissue engineering [48]. Additionally, biologic agents and cellular therapy can be employed to enhance osteogenic, osteoconductive and/or osteogenic potential of augmentation procedures.

#### 3.1.1. Alveolar Ridge Preservation

Following tooth extraction, a local inflammatory response predominates after blood clots within the socket. In the first week, endothelial cells proliferate to restore the soft tissue integrity. New bone formation can be observed as early as at two months and continues up to six months post-extraction. Without masticatory forces on the periodontium, resorption of the alveolar bone occurs in both horizontal and vertical dimensions, leading to invagination of overlying soft tissues. Most statistically significant reduction of alveolar bone occurs during the first month [84,85].

The purpose of alveolar ridge preservation after tooth extraction is to minimize or prevent resorptive bone remodeling and to maximize bone and/or soft tissue availability before the placement of a definitive prosthetic restoration. In the esthetic zone of non-molar areas, changes in the buccal bone and soft tissues are of high concern [84]. Socket grafting and socket sealing are examples of treatment modalities that use biomaterials and barrier materials to fill the extraction socket by primary or secondary intention healing. In comparison to natural socket healing without intervention, socket grafting with bone substitute materials with or without socket sealing with a barrier membrane was superior in preventing horizontal and vertical bone resorption and improved successful implant placement without additional bone grafting at the time of re-entry [84,86,87]. Histologically, higher new bone content was observed in sockets with alveolar ridge preservation after extraction versus natural healing [88].

Among natural socket grafting materials, a composite graft of xenogeneic and allogeneic bone materials covered by a collagenous barrier showed the highest preventive effect in changes of horizontal dimension and height [86,87]. In preserving ridge width, Iocca et al. showed that autologous bone marrow, followed by FBDA plus membrane, achieved the greatest success. Early exposure of the membrane will compromise the effectiveness of guided tissue regeneration [87].

For synthetic filler materials, bioabsorbable PLGA sponges showed histological evidence of well-structured mature bone formation and complete remodeling without the presence of grafting particles at six months after ridge preservation. In comparison to spontaneous healing, less bone resorption was observed with adequate bone quality suitable for implant insertion [89]. These findings are significant that particles of FBDA, deproteinized bovine bone material (DBBM), and bioactive ceramics require longer time to integrate fully as graft particles were found at 6-9 months after insertion. In one canine study by Salamanca et al. (2014), hydroxyapatite/*β*-TCP ceramic mixed with homogenous collagen solution showed slightly higher success in new lamellar bone formation and reabsorption in addition to comparable osteoconductivity to bovine-derived bone (Bio-Oss^®^) plus collagen membrane [90].

Regardless of which biomaterials are employed, bone resorption is not totally prevented and some loss in ridge width and height is expected [84]. High heterogeneity in healing patterns was also observed with socket grafting and can be possibly explained by tooth type, presence or absence of adjacent teeth, level of bone at adjacent teeth, and number of roots and socket morphology of extracted tooth. In addition, the method to measure dimensional changes may contribute to this heterogeneity [84]. Therefore, no definitive conclusions can be drawn regarding which biomaterial is superior [84,88].

#### 3.1.2. Vertical and Horizontal Ridge Augmentation

Guided bone regeneration (GBR) with a membrane is the most frequently employed surgical technique to regenerate atrophic residual ridges and operates on the triad of biological principles: (1) cell occlusivity, (2) wound stabilization, and (3) space making and maintenance. In comparison to other surgical techniques such as distraction osteogenesis, bone inlay, or block grafting, GBR achieved the highest reliability in achieving vertical bone gain with the lowest complication rate and minor overall resorption [91,92]. The main complication was membrane exposure. Moreover, greater defect reduction was observed in using non-exposed barrier membranes [93,94]. Several different combinations of surgical modalities in GBR may be used to treat bony defects (i.e., fenestration, dehiscence, vertical defects, horizontal defects) and to obtain adequate bone volume for staged or simultaneous implant placement.

Resorbable polymeric membranes are preferred in predictably regenerating non-critical-size defects, improving soft tissue healing and cost-effectiveness, and lowering surgical stress and complications. To improve their mechanical stability and space-maintaining ability, fixation screws and tenting screws can be placed, respectively. In comparison, non-resorbable membranes, like those including a titanium framework, perform more reliably in regenerating larger defects for their intrinsic space-making properties and controlled barrier effect over time. Membrane porosity plays an important role in directing angiogenesis and the proliferation of bone progenitor cells over the competing soft tissue cells [71].

The usage of composite grafts consisting of bone allografts or xenografts with a resorbable membrane showed comparable clinical outcomes to autologous bone grafts [75,93,95,96,97]. In the staged approach of GBR prior to implant placement, the combination of particulate xenograft, autologous bone and resorbable membrane achieved the maximum bone width gain; in the simultaneous approach, the combination of particulate xenograft with a resorbable membrane was most frequently used and achieved significant reduction in defect height changes [94]. The use of autologous bone and bone substitute materials together can (1) combine osteogenic and osteoinductive properties of autografts and osteoconductive properties of bone substitute materials, and (2) reduce the total harvested autologous bone volume [98].

These results support that composite grafts can serve as a good alternative that overcomes the major complications associated with autogenous bone harvesting such as donor site morbidity and limited supply of autogenous bone. Differences in surgical techniques and experience of operator should be considered in calculating clinical outcomes of using different combinations.

#### 3.1.3. Maxillary Sinus Augmentation

**Figure 2:**
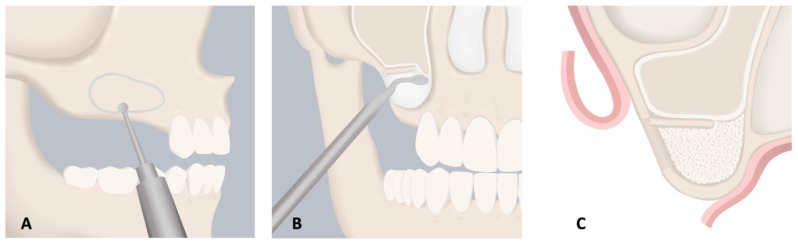
Lateral Window Approach for Maxillary Sinus Augmentation. (**A**) After the full thickness mucoperiosteal flap is raised, the outline of the lateral window is marked with a round bur or piezoelectric surgical tip. (**B**) Before elevating the sinus membrane, the buccal bone is either removed or pushed inward to gain access to the Schneiderian membrane. The membrane is carefully elevated using blunt instruments. (**C**) The sinus compartment is filled with grafting material and covered with resorbable barrier membrane, which can consist of polymeric scaffolds. Reprinted from [99] with permission from Elsevier.

**Figure 3:**
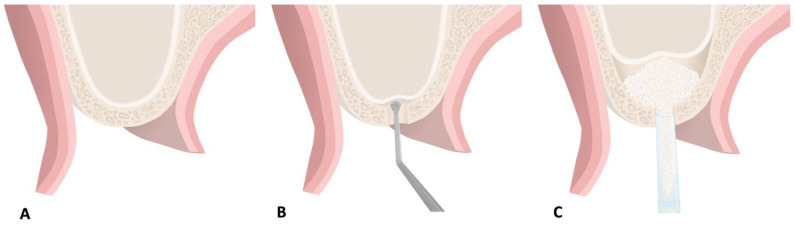
Transalveolar Approach for Maxillary Sinus Augmentation. (**A**) A full thickness mucoperiosteal flap is raised on the edentulous ridge. (**B**) After marking the location of the future implant, the site is prepared with implant drills to approximately 1.0–1.5 mm below the sinus floor. Osteotomes are used to fracture the sinus floor and elevate the membrane. (**C**) The sinus compartment is gradually filled with grafting material until the appropriate depth for implant placement is achieved. Reprinted from [99] with permission from Elsevier.

Maxillary sinus augmentation can be achieved via lateral window technique or transcrestal approach with the purpose of creating a space between the sinus floor and Schneiderian membrane to fill with biomaterials that promote new osseous tissue formation (Figure 2 and Figure 3) [99]. A variety of autologous bone, and allogeneic, xenogeneic, or alloplastic bone substitute materials have shown success in achieving the desired outcome of increased vertical bone height for future implant placement.

In a systematic review by Al-Nawas et al., no statistically significant differences were observed in implant survival among bone autografts and bone substitute materials [96]. Theoretically, the superior osteogenic and osteoinductive capacities of autogenous bone could be beneficial in short-term healing. Clinically, no significant differences in new bone formation were observed in using allogeneic, xenogeneic, or synthetic bone substitutes with or without autogenous bone [67,96,100]. Possible clinical considerations of usage of bone substitutes over autografts include reducing invasiveness of surgery and surgical time [67]. Similarly, a histomorphometric analysis revealed that though higher mineralized bone was evidenced in early healing for autologous bone, total bone volume after 9 months appeared comparable with using bone substitute materials [101]. Conflicting findings exist in regard to comparing healing periods between these two groups and if the success of the maxillary sinus augmentation is dependent on the graft materials used [96]. Overall, regardless of which biomaterials are used, maxillary sinus augmentation is safe and well-tolerated by patients [100].

The success of maxillary sinus augmentation is heavily indicated by anatomic differences of the sinus cavity rather than which graft material is used. New bone can be predictably generated only in narrow sinuses with at least two walls contacting the grafting material. This is possibly explained by the innate osteogenic potential of sinus walls, sinus floor and Schneiderian membrane when in contact with grafting material [102].

#### 3.1.4. Temporomandibular Joint Reconstruction

TMJ consists of two articulating anatomic components: the temporal bone and the mandibular condyle. The condylar fibrocartilage is covered by a dense fibrous layer and consists of cellular layers that contains collagen type I primarily, minimal amounts of collagen types II, III, IV, IX, and X, and glycosaminoglycans (aggrecan and versican) [103]. The articulating disc, which is attached to the temporal bone and the condyle, is also rich in collagen type I but lacks inherent vascularization or innervation. The non-collagenous ECM of both the fibrocartilage and disc are made of dermatan sulfate and chondroitin sulfate-based proteoglycans [104].

A degenerative disease process of the TMJ can involve the disc, fibrous tissue covering, proliferative and hypertrophic layers of the fibrocartilage, or condylar bone. If non-invasive and minimally invasive treatments are ineffective in improving symptoms or function of the joint, partially or totally replacing the TMJ should be considered [105]. Currently, the consensus of treatment modalities indicates a reconstructive procedure with autologous tissues in young patients and metallic prostheses in adult patients are preferred [106]. However, these conventional strategies fail to provide long-term efficacy in forms of poor condylar remodeling, continuous erosion of the articular surfaces, or osteophyte formation [104].

To achieve better clinical outcomes and long-term prevention of ossifications and adhesions, regenerative TMJ therapies have been proposed. Current methods to regenerate the TMJ involve biphasic cartilage and bone engineering with ex-vivo cell seeding and bioactive molecules on an acellular scaffold. Of many multipotent cell types that were tested for condylar cartilage regeneration, DPSCs have been well-documented for their high availability and multi-lineage proliferation into chondrogenic cells, osteoblasts, and other crucial cell types [103,104]. For disc regeneration, dermal fibroblasts induced with insulin-like growth factor 1 (IGF-1) have shown success in their high availability and chondrogenic potential. Other growth factors of interest include transforming growth factor-βeta 1 (TGF-β1), FGF, and PDGF; their oncogenic potential in the context of craniofacial tissue regeneration require further investigation [104].

For TMJ disc regeneration, natural materials like collagen, fibrin, chitosan, and dECM sheets have been widely used [107]. These natural products possess similar mechanical properties to the native disc such as cell adhesion and infiltration, cell proliferation, and proteoglycan deposition [103,108].

Synthetic polymers have been proposed to produce artificial ECM for TMJ fibrocartilage regeneration with superior mechanical strength and biodegradability to natural materials [109]. One example that is approved by the FDA for clinical usage is poly-L-lactic-co-glycolic acid (PLGA). PLGA is effective at chondrogenesis by promoting colonization and proliferation of mesenchymal stem cells and interacting with chondrocytes and other TMJ discal cells [110]. PCL fibers is a type of pro-regenerative biomimetic nanofiber that is also approved by the FDA for clinical applications [111]. After being processed by electrospinning, successful results for osteochondondral regeneration were reported. In addition, PCL has recently gained popularity in craniofacial reconstruction for its excellent biocompatibility and low degradation rate [104,108,111,112].

For biofabrication methods, 3D printing provides an effective method to produce personalized prostheses with a spatiotemporal delivery of bioactive molecules and cells for tissue regeneration [111]. Living cells can be employed within the fibers during the manufacturing stage or be seeded onto the matrix for colonization. The ability to tune 3D-printed scaffolds to achieve optimal biomechanical properties and mimic natural ECM makes 3D printing has been evidenced to positively impact the performance of their implantation [104,108,112]

### 3.2. Periodontal Surgery

#### 3.2.1. Periodontal Regeneration

Periodontal regeneration aims to regenerate alveolar bone, periodontal ligament and cementum around teeth affected by periodontitis. Periodontal regeneration using guided tissue regeneration (GTR) allows selecting bone cells, fibroblasts, and PDL cells to populate the periodontal wound. In 1976, Melcher developed the concept of using barrier membrane to guide the biological process of wound healing. More specifically, these membranes exclude epithelial cells from infiltrating into the bony defects [113]. Any combination of bone fillers, membranes, and biologics can be directly administered in the defect to regenerate new alveolar bone, cementum and PDL [72]. The ideal properties of membrane should respect several key principles: (1) biocompatibility, (2) cell exclusion, (3) space maintenance, and (4) clinical handling [71,72].

Historically, non-resorbable polymeric membranes such as polytetrafluoroethylene (PTFE) were used for guided bone regeneration. However, due to their rigidity, these membranes are rarely used in periodontal regenerative surgery because the use of minimally invasive surgical techniques does not allow for predictable barrier membrane insertion and adaptation. Instead, resorbable membranes made from collagen are indicated. In addition, the chemical bonds between collagen membrane can be reinforced through cross-linking, which lead to slower resorption time and decrease in risk of membrane exposure in the oral cavity and potential for complication such as bacteria infiltration and graft contamination.

**Figure 4:**
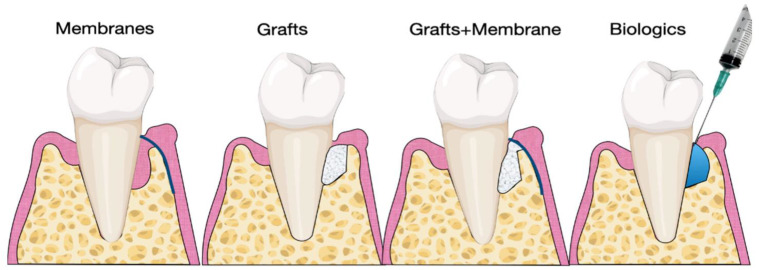
Biomaterials for Periodontal Regeneration. Reprinted from [74] with permission from MDPI.

Bioabsorbable scaffolds like collagen membranes have been developed to avoid the second surgical trauma to the healing process associated with non-resorbable membranes. The usage of regenerative biomaterials in GTR is well-documented (Figure 4) [114]. A systemic analysis of outcomes of GTR with a collagen membrane demonstrated that pocket depth reduction is predictably achieved with or without a bone substitute [115].

Scaffolding materials are typically applied to defect sites in addition to membranes. The ideal properties of graft material for periodontal include (1) osteoconduction, (2) osteoinduction, and (3) osteogenicity. However, only autogenous materials from patients have all three properties. In contrast, polymeric scaffold materials are typically osteoconductive, providing space maintenance to enable cells to migrate into the defect site. Since the 1980s, various polymeric matrices and scaffolds have been used for periodontal regeneration of intraosseous defects.

Similarly, 3D-printed scaffolds have been recently developed to improve upon the existing supporting matrices. In contrast to the brittleness, poorly processed porosities, and generic structures of the conventional grafts, 3D scaffolds can be tailored to the specific needs of patients [48,116]. Compartmentalization, internal topographies, and pore sizes and angulations can be designed with precision to optimally regenerate each tissue type of the periodontium [116,117]. Cell therapy can be employed within the scaffold architecture via two methods: (1) cell seeding into a pre-made scaffold, and (2) cell encapsulation during scaffold fabrication in the form of biodegradable hydrogel polymer matrix [48]. A case report demonstrated that the use of 3D printed PCL scaffold for periodontal regeneration. However, the graft failed due to slow degradation rate of PCL compared to surrounding tissue, which resulted in graft exposure [48].

Recent advances in additive manufacturing technology allow for the fabrication of nanoscale scaffolds with controllable properties including fiber diameter, porosity, morphology, and surface characteristics [118]. More specifically, electrospinning utilizes polymeric solution to generate nanofibrous scaffolds with high surface area to volume ratio, enhanced protein absorption, activation of specific gene expression and intracellular signaling to potentiate cell behavior towards regeneration. Nano-composite electrospun fibers can be manufactured by blending various polymers and functional components together. These scaffolds have the ability to natural ECM to improve cell survival, attachment and organization by promoting protein absorption, activating specific gene expression and intracellular signaling pathways [118]. Various additives can be incorporated into these electrospun constructs including bioceramics, carbon-based components, metal components to enhance the scaffold’s physical-chemical-biological properties and regenerative capabilities. In addition, growth factors, proteins and drugs can be incorporated into these polymeric matrices to regular cellular reactions and tune local inflammatory microenvironment to promote periodontal regeneration [118]

Systematic reviews and randomized controlled clinical trials provide evidence that the combined use of EMD and human recombinant platelet-derived growth factor (rhPDGF-BB) with beta-tricalcium phosphate can provide regenerative results comparable to bone graft materials [119]. Future research and clinical translation are required to make polymeric materials predictable for periodontal regeneration.

#### 3.2.2. Periodontal and Peri-Implant Soft Tissue Regeneration

Soft tissue grafting around natural teeth and dental implants have been increasingly used in clinical practice since its introduction in the 1960s [120,121,122]. The main goals of periodontal and peri-implant plastic surgery are to augment tissue thickness and width, correcting mucogingival deformities, improve esthetics in patients with gingival recession or lack of keratinized tissue [123]. Although the use of autogenous soft tissue grafts is considered the gold standard for achieving complete root coverage and adequate soft tissue augmentation, patient morbidity has been reported as one of the major shortcomings of an autologous soft tissue graft harvesting procedure [124].

**Figure 5:**
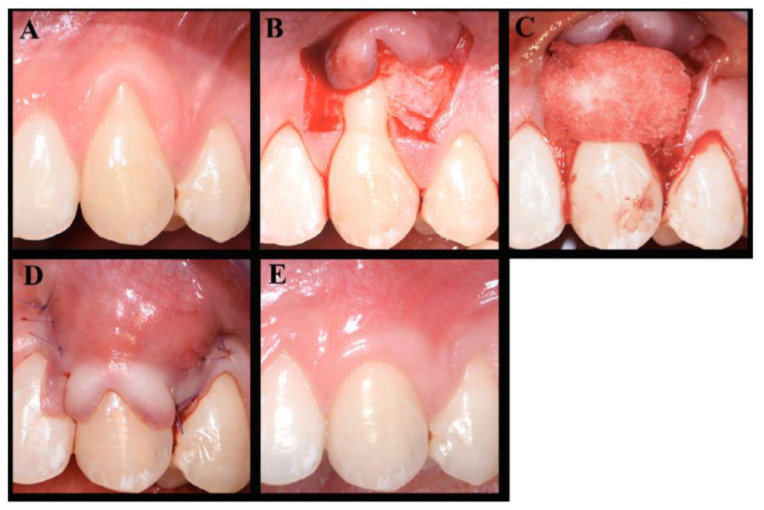
Volume-Stable Collagen Matrix for Periodontal Plastic Surgery. (**A**) Gingival recession defect on a maxillary canine (**B**) A split-full-split flap limited to the canine was performed (**C**) After the de-epithelialization of the anatomical papillae, a volume-stable collagen matrix was applied on the root surface and sutured to the de-epithelialized papillae (**D**) The flap was coronally advanced and sutured (**E**) One-year outcomes. Reprinted from [125] with permission from Wiley.

Extracellular matrix scaffold biomaterials have gained significant popularity for periodontal and peri-implant soft tissue augmentation in the recent years. The main advantages of ECM scaffolds compared to autogenous grafts harvested from the patient’s palatal donor site include material availability, avoidance of surgical harvest of donor tissue, reduction of surgical time and patient preference (Figure 5) [125]. Current scaffolds used can be classified based on their origin including allogenic, xenogeneic, alloplastic, and living cell constructs with their respective advantages and drawbacks. The ideal properties of such scaffolds include (1) biocompatibility, (2) space maintenance, (3) blood clot stabilization, (4) promotion of cellular migration and proliferation, (5) ease of manipulation during surgical procedure, (6) ease of adaptation and positioning to surgical site [125].

**Figure 6:**
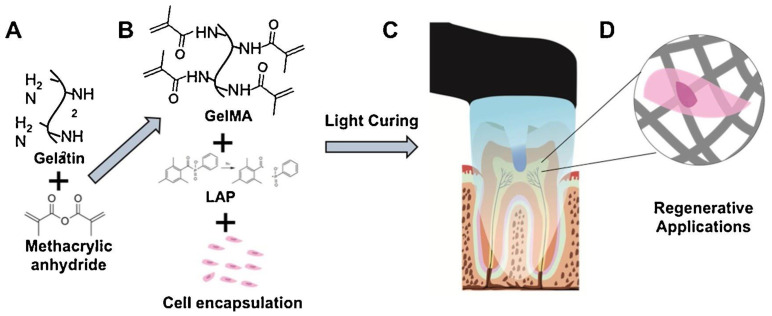
Photopolymerizable Cell-Laden Gelatin Methacryloyl Hydrogels for Regenerative Endodontics. Example of application of GelMA hydrogel in regenerative dentistry. (**A**) Synthesis of GelMA macromer (**B**) Cell encapsulation (**C**) Example intracanal hydrogel loading and photopolymerization (**D**) The resulting cell-laden hydrogel material. Note that although the schematic depicts an example for regenerative endodontics, the material can be used for any application of intra-oral regeneration, such guided periodontal regeneration, alveolar bone growth and others. Reprinted from [145] with permission from Elsevier.

Several alternative graft materials are used by clinicians including natural and cadaveric scaffolds and polymeric matrices. Natural and cadaveric scaffolds include decellularized human dermis and human amniotic membrane, which can promote cellular migration and revascularization [126,127,128]. Other ECM scaffolds include xenogeneic collagen matrices which includes bilayered collagen matrix, volume-stable collagen matrix and xenogeneic acellular dermal matrix which can support the proliferation of fibroblasts and keratinocytes (Figure 6) [128,129,130]. In addition, biologics including EMD, PDGF, platelet concentrates and FGF-2, can be applied to these ECM scaffolds to promote regeneration [125].

Polymeric matrices are widely used as biomaterials in tissue engineering and regenerative medicine for scaffold fabrication [131]. As these materials are devoid of cells and signaling molecule, their primary purpose is space maintenance to allow for fibroblast and keratinocyte migration and proliferation [125]. Polymeric matrices have shown good potential for drug delivery and may be useful in the context of periodontal plastic surgery for biologics delivery. However, there is limited evidence on the use of synthetic polymeric biomaterials including PCL, PLGA and PLLA for periodontal and peri-implant soft tissue augmentation in humans as a stand-alone scaffold because they do not potentiate cell function towards new tissue formation or neovascularization. Future research should explore the combination of polymeric scaffolds in combination with biologics.

### 3.3. Regenerative Endodontics

Polymeric scaffolds have been used in Regenerative Endodontic Procedures (REP) to provide a suitable physiological environment for biologically replacing damaged dentin-pulp complex and root structures. In the endodontic literature, regeneration can also be referred to as revascularization or revitalization [132,133]. The main goals of regenerative endodontics are to close the root apex, increase root length, thicken root canal walls, and achieve pulp regeneration, all while maintaining biocompatibility. REP was originally developed to treat immature necrotic teeth, but recently, they have also been performed on necrotic permanent teeth, vital mature permanent teeth, and resorbed teeth with a history of trauma [134].

The scaffold reported to be used the most during REP is blood clot. This technique generally involves canal preparation and disinfection, followed by induction of blood clot from the periapical region. However, there is an increasing number of scaffolds that have showed to be clinically successful, namely platelet-rich plasma (PRP) scaffolds, platelet-rich fibrin (PRF) scaffolds, collagen membranes, collagen-hydroxyapatite scaffold, collagen-gelatin hydrogels with and without fibronectin, chitosan hydrogels with and without microparticulate dentin, alginate-laponite hydrogels incorporated with DPSCs and VEGF, angiogenic hydrogels, gelatin methacryloyl (GelMA) hydrogels with and without human DPSCs, and GelMA hydrogels with and without odontoblast-like cells and endothelial colony forming cells.

Some of the scaffolds that allowed for continued root formation, such as apical closure, increased root length, and thickened root canal walls, include PRF scaffolds, PRP scaffolds, collagen membranes, and collagen-hydroxyapatite scaffold, known as SynOss putty. PRF scaffolds have shown evidence of apical closure, resolution of apical radiolucency, continued root lengthening, and thickening of dentinal walls in immature permanent teeth with necrotic pulps [135,136,137,138]. Similarly, PRP scaffolds showed the same outcomes as PRF scaffolds, with no statistically significant differences between the two [137,139]. Bio-Gide collagen membranes (Geistlich, Wolhussen, Switzerland) have shown to promote the development of dentinal wall in the middle third of the root, thus reinforcing the root to prevent cervical root fractures [140]. SynOss putty used with blood as scaffold had contradictory findings. One study showed that the use of SynOss putty in combination with blood as scaffold in REP lead to the formation of an intracanal mineralized tissue that solidified with the newly formed cementum-like tissue on dentinal walls, essentially improving the integrity of immature non-infected human teeth [141]. However, another study showed that there was no tissue regeneration present in the non-infected ferret teeth samples using SynOss putty as scaffold [142].

Polymeric scaffolds are also used to improve the biological performances of the REP, and can influence cell spreading, proliferation, release, recruitment, viability, and degradability. These include GelMA hydrogels with and without additional cells, injectable HA hydrogels, alginate-laponite hydrogels, collagen-gelatin hydrogels, chitosan hydrogels, and chitosan-based scaffolds. Higher-stiffness GelMA hydrogels seeded with odontoblast-list cells (OD21) are shown to have higher spreading, proliferation, and viability near dentinal walls. Similarly, endothelial colony forming cells (ECFC) incorporated on stiffer GelMA hydrogels exhibited higher spreading and a tendency to form endothelial monolayers with active angiogenic sprouts in fabricated microchannels [143]. In addition, GelMA microspheres laden with hDPSCs showed capability to support multiple cell functions as well as cryopreservation of hDPSCs in vitro and showed even better degradability and pulp tissue regeneration in vivo compared to bulk GelMA hydrogels [144]. Photopolymerizable cell-laden GelMA hydrogels have been developed to be light cured using dental curing light (Figure 6) [145]. Injectable HA hydrogels reinforced with platelet lysate (PL) have shown to promote hDPSC recruitment and stimulate chemotactic and pro-angiogenic activity in both in vivo and ex vivo models [146]. There are many more scaffolds that have been designed to support other cell functions, such as differentiation and mineralization of human apical papilla cells (hAPC), while also enhancing antibacterial properties and providing dentinal disinfection [147].

Scaffolds in REP have also mainly been used to achieve pulp regeneration, which often translates into elimination of clinical signs and symptoms and regain of pulp sensibility. Such scaffolds include PRF membranes, alginate-laponite hydrogels, human-derived composite amnion-chorion membrane (ACM), collagen-gelatin hydrogels, and soft angiogenic biomimetic acellular peptide hydrogels. One study showed that teeth that have been endodontically treated using PRF membranes showed elimination of all clinical signs and symptoms, and presence of tooth sensibility 12 months after the initial treatment, which is indicative of the formation of a vital pulp-like tissue [148]. Alginate-laponite hydrogels encapsulating DPSC and VEGF have shown to promote a sustained release of VEGF and thus allow for revascularization and regeneration of pulp-like tissue in vivo [149]. Human composite ACM is a scaffold that contains a combination of growth factors and cytokines that can facilitate the controlled recruitment of progenitor stem cells and thus enhance pulp regeneration. In fact, when compared to using a blood clot alone or using a blood clot with collagen membrane for pulp regeneration in mature teeth, ACM produced more intracanal fibrous tissue and odontoblast-like cell lining [134]. Finally, angiogenic hydrogels have similar material properties to that of the native dental pulp and can promote ideal biointegration and soft tissue regeneration. They have been shown to re-establish vasculature in dental root canals [150].

Although there has been many studies highlighting the success of the existing REP scaffolds, many have their limitations. For one, many membranes are still in their original phases and being tested in vitro. For these newer scaffolds, the successful results obtained from in vitro assays will form the basis for future studies as they will require more thorough in-vivo analysis of their effectiveness, as well as further preclinical and clinical studies [134,143]. For another, many studies showed a negative response to pulp vitality assessments during the follow-up period despite demonstrating apical closure and resolution of the periapical lesion on radiographic examination. To regain nerve function after REP takes a long time. Therefore, a longer follow-up period may be required for pulp sensibility tests to display a positive result [138,139].

### 3.4. Whole Tooth Regeneration

Dental conditions such as dental caries or periodontal disease, whether with or without dental treatment, ultimately lead to loss of the affected teeth with time. Although the completely edentulous population is reducing over the past decades, 2.3% of the global population remains edentate in 2010, representing 158 million people [151]. Tooth loss is associated with systemic health conditions such as malnutrition, hypertension, and obesity [152]. There is a wide range of treatment options, ranging from restorations, prostheses, or dental implants. With the emergence of tissue engineering, treating edentulism with bioengineered teeth is coming to the horizon of researchers and clinicians. These innervated and vascularized vital teeth would mimic closely the appearance and function of natural dentition, offering a better outcome than any of the existing synthetic dental implants to date.

Biodegradable scaffold materials are used to support tooth formation into the desired size and shape. Post-natal tooth buds are seeded into the scaffold and transplanted into renal capsules for maturation. In 2002, Young et al. seeded single-cell suspension from porcine third molar tooth tissue PLGA scaffolds, and successfully formed recognizable tooth structures such as dentin, odontoblasts, enamel and well-defined pulp chamber [153]. The promising results opened the door to studying scaffold-based techniques in whole tooth regeneration. The same scaffold was used by Duailibi, where 4-day postnatal rat tooth bud cells seeded for 1h generated tooth tissues most reliably [154]. Smith et al. explored GelMA hydrogels as scaffolds for postnatal dental cells. The generated tooth buds contained biomarkers characteristic of a natural tooth bud [155]. Although the experiments produced teeth-like structures, the size of the teeth was too small, and the shape was uncontrollable. Until now, the closest to a real-size tooth was achieved by combining adult dental cells with decellularized natural tooth bud ECM scaffolds. Six months after implantation into mini pig hosts, organized dentin and enamel-like tissues were observed, comparable to natural teeth [156]. Several studies utilize embryonic or induced pluripotent stem cells based scaffold free strategies for tooth bioengineering [157,158,159].

Several challenges remain in the tissue engineering field, preventing the clinical application of whole tooth regenerative therapy. Controlling the size and shape of the bioengineered tooth is a significant concern. To have optimal functionality, the regenerated teeth need to have a precise crown shape for occlusion. To date, no scaffold can direct the tooth generation with such precision. Another challenge is the integration of the engineered tooth to the host supporting tissues such as alveolar bone and PDL. Vascularization and innervation are especially hard to obtain but essential for the longevity of the teeth. Moreover, the population needing whole tooth regenerative therapy would have lost their natural dentition due to underlying conditions such as caries and periodontal disease. These oral conditions further complicate the situation as they put the patient at higher risk of infection and having an unhealthy oral environment.

The future is promising for whole tooth bioengineering. With fully functional tooth roots, pulp and attachment apparatus, the crown can potentially be 3D printed for better size and shape accuracy [160]. Although researchers are optimistic about achieving successful whole tooth regeneration, the cost must be taken into consideration to make its clinical application possible.

### 3.5. Salivary Gland Regeneration

The salivary system comprises the parotid, submandibular, and sublingual glands, and ~1000 minor glands in the oral mucosa. Salivary glands (SGs) are composed of two types of secretory acinar cells (fluid-secretory epithelial cells) and ductal cells forming the duct network to secrete saliva. This parenchymal tissue is surrounded by myoepithelial and endothelial cells. When SGs are damaged, frequently in patients treated for radiotherapy for head and neck cancer and patients with Sjögren’s syndrome, they experience a decrease in saliva production due to the loss of acinar cells function. Consequently, patients experience dry mouth, difficulty swallowing, oral infection, tooth decay, taste loss, and malnutrition.

Several strategies have been developed to study and culture SG cells in vitro using different hydrogels compositions. Nam et al., used fibrin- and laminin-based hydrogels to promote the regeneration of salivary tissue. The authors found that chemically conjugated fibrin with laminin peptides applied to wounded mouse submandibular glands in vivo and promoted the cell organization into salivary tissue, indicating that damaged salivary gland tissue can grow and differentiate using these hydrogels as a 3D scaffold [28,29]. In another study using trimers laminin- I II conjugated with fibrin hydrogels (LIP-T-FH), the hydrogel was able to increase the expression of acinar differentiation markers and elevate saliva secretion on Par-C10 acinar cells and C57BL/6 mice. The LIP-T-FH significantly increased the expression of the acinar cell differentiation markers and saliva secretion compared with monomeric form [161].

**Figure 7:**
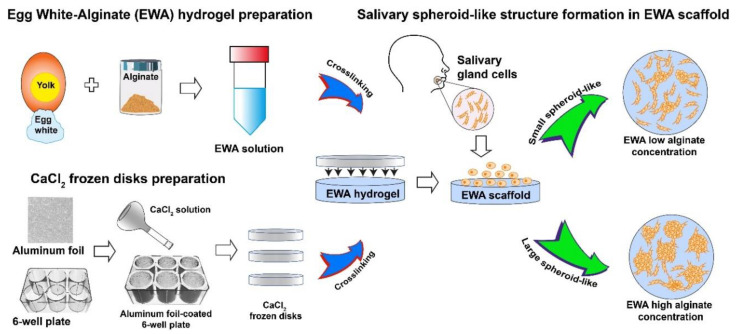
Egg White Alginate (EWA) hydrogel preparation for salivary gland spheroid-like structure formation. EWA is a novel hydrogel which combines the advantages of both egg white and alginate. The egg white material provides extracellular matrix (ECM)-like proteins that can mimic the ECM microenvironment, while alginate can be tuned mechanically through its ionic crosslinking property to modify the scaffold’s porosity, strength, and stiffness. Reprinted from [163] with permission from MDPI.

Ozdemir et al., synthesized HA-based hydrogels with different polymer concentrations and thiol/acrylate ratios. In hydrogels with a G′ ≤ 216 Pa and a thiol/acrylate ratio ≥ 18, salivary human stem cells self-assembled into acini-like structures, with an average diameter of 50 μm, and the spheroid size and size distribution were dependent on the HA content in the hydrogel [162]. Placenta basement membrane extract (PBME) promotes the polarization and organization of SG cells in 3D hydrogels. Maria et al. used fibronectin- and PBME gels to expand and differentiate primary human salivary gland epithelial cells (huSGs) in a serum-free medium. The same group has recently grown human salivary spheroids using a hydrogel made of egg white and alginate (Figure 7) [163]. These systems allowed the morphological and functional differentiation of salivary ductal cells into acinar-like cells, exhibiting a polarized acinar 3D units or monolayers with tight junction proteins, acinar proteins, and acinar adhesion-related cell markers [164].

## 4. Future Directions

Additive manufacturing (AM) is one of the most promising technologies aiming to construct 3D functional organs in vitro based on a layer-by-layer building-up process from a 3D CAD design [165]. Different AM techniques are available to achieve 3D geometrically complex structures that can be used for tissue regeneration and dental applications, including stereolithography, digital light processing, inkjet, material extrusion [24]. Even when some successful 3D models have been created using AM process, the materials remain as “static scaffold”, supporting cell growth and development but maybe not enough to support tissue or organ development [166].

It is well known that a biological system, besides its complexity, possesses a dynamic environment under constant reorganization to adapt to external factor facilitation and ensure the excellent functionality of organs and cells [166]. 4D printing is a new manufacturing concept that involves the use of 3D printed structures made with responsive polymers that can be stimulated by external factors such as pH, humidity, light, and temperature. These materials allow dynamic responses to in vivo conditions by changing their shape or color, producing an electrical stimulus, becoming bioactive, self-assembling, or performing an intended function [166,167]. The 3D structure generated represents a more realistic model mimicking the native human environment and ensuring the good performance of living entities.

Although responsive materials have been studied for years, only a few have been used in 4D printing for tissue engineering [166,167]. As mentioned above, the material’s requirements for tissue engineering are strict to guarantee the correct biological performance. An ideal polymer matrix must be biocompatible, biodegradable, present mechanical strength, bioactive, and fit the dynamic environment that living organisms have. Many potential applications of smart hydrogels along with 3D/4D printing exist in craniofacial and tissue regeneration leading, for instance, the self-assembling, self-memory material, self-repair, controlled release of drugs and biomolecules, of 3D polymeric matrices, which may offer a pivotal advantage in the development of in vitro tissue and organs [2].

## 5. Conclusions

Despite advances in polymeric scaffolding materials used to treat dental, oral, and craniofacial deficiencies, true regeneration of tissues that combines native morphology, physiologic function and esthetic remains a challenge in the field of DOC regenerative medicine. Further research is needed to develop polymeric biomaterials with tunable mechanical properties that potentiate cell function, matching degradation rate similar to physiological remodeling processes, surface functionalization with gene vectors or biologics to enhance cell interactions, and ability to be used in additive manufacturing including 3D and 4D bioprinting. In the future, polymeric scaffolds will play a significant role in personalized patient care to ultimately provide predictable treatment options to enhance clinical outcomes and patient quality of life.

## Data Availability

Not applicable.
